# Body Mass Index and Disease Activity in Chronic Inflammatory Rheumatic Diseases: Results of the Cardiovascular in Rheumatology (Carma) Project

**DOI:** 10.3390/jcm10030382

**Published:** 2021-01-20

**Authors:** Jesús A. Valero-Jaimes, Ruth López-González, María A. Martín-Martínez, Carmen García-Gómez, Fernando Sánchez-Alonso, Jesús T. Sánchez-Costa, Carlos González-Juanatey, Eva Revuelta-Evrad, César Díaz-Torné, Cruz Fernández-Espartero, Carolina Pérez-García, Vicenç Torrente-Segarra, Ginés Sánchez-Nievas, Trinidad Pérez-Sandoval, Pilar Font-Ugalde, María L. García-Vivar, Elena Aurrecoechea, Olga Maiz-Alonso, Ramón Valls-García, José A. Miranda-Filloy, Javier Llorca, Santos Castañeda, Miguel A. Gonzalez-Gay

**Affiliations:** 1 Division of Rheumatology, Hospital Universitario de Donosti, 20014 San Sebastián, Spain; jesval1204@gmail.com (J.A.V.-J.); OLGA.MAIZALONSO@osakidetza.eus (O.M.-A.); 2 Division of Rheumatology, Hospital Universitario Hospital General Virgen de la Concha, 49022 Zamora, Spain; lopezgonzalez.ruth@gmail.com; 3 Research Unit of Spanish Society of Rheumatology, 28001 Madrid, Spain; xily_mm@yahoo.com (M.A.M.-M.); fernando.alonso@ser.es (F.S.-A.); jesus.sanchez@ser.es (J.T.S.-C.); 4 Division of Rheumatology, Consorci Sanitari de Terrassa, Terrassa, 08191 Barcelona, Spain; ggcarme@gmail.com; 5 Division of Cardiology, Hospital Universitario Lucus Augusti, 27003 Lugo, Spain; carlosjuanatey@secardiologia.es; 6 Division of Rheumatology, Hospital General Universitario de Ciudad Real, 13005 Ciudad Real, Spain; evaevrard19@gmail.com; 7 Division of Rheumatology, Hospital de la Santa Creu I Santa Pau, 08041 Barcelona, Spain; cesardiaztorne@gmail.com; 8 Division of Rheumatology, Hospital Universitario de Móstoles, 28935 Madrid, Spain; mcruzespartero@yahoo.es; 9 Division of Rheumatology, Hospital del Mar, 08003 Barcelona, Spain; perezgarcia.carolina@gmail.com; 10 Division of Rheumatology, Hospital Comarcal Alt Penedès Garraf, 08720 Barcelona, Spain; vtorrente@csap.cat; 11 Division of Rheumatology, Complejo Hospitalario Universitario de Albacete, 02006 Albacete, Spain; giness@sescam.jccm.es; 12 Division of Rheumatology, Complejo Asistencial Universitario de León, 24071 León, Spain; trinidad.perezsandoval@gmail.com; 13 Division of Rheumatology, Hospital Universitario Reina Sofía, 14004 Córdoba, Spain; fougp@hotmail.com; 14 Division of Rheumatology, Hospital Universitario Basurto, 48013 Bilbao, Spain; marialuz.garciavivar@osakidetza.eus; 15 Division of Rheumatology, Hospital de Sierrallana, 39300 Santander, Spain; elena.aurrecoechea@scsalud.es; 16 Division of Rheumatology, Hospital Universitario de Palamós, 17230 Barcelona, Spain; g2179rvg@gmail.com; 17 Division of Rheumatology, Hospital Universitario Lucus Augusti, 27003 Lugo, Spain; jose.alberto.miranda.filloy@sergas.es; 18 University of Cantabria and CIBER Epidemiología y Salud Pública (CIBERESP), 39005 Santander, Spain; javier.llorca@unican.es; 19 Division of Rheumatology, Hospital Universitario de la Princesa, IIS-Princesa, EPID-Future, Universidad Autónoma de Madrid (UAM), 28006 Madrid, Spain; scastas@gmail.com; 20 Division of Rheumatology, Hospital Universitario Marqués de Valdecilla, University of Cantabria, 39008 Santander, Spain; 21 Epidemiology, Genetics and Atherosclerosis Research Group on Systemic Inflammatory Diseases, Rheumatology Division, IDIVAL, 39011 Santander, Spain; 22 Cardiovascular Pathophysiology and Genomics Research Unit, Faculty of Health Sciences, School of Physiology, University of the Witwatersrand, Johannesburg 2050, South Africa

**Keywords:** body mass index, obesity, rheumatoid arthritis, ankylosing spondylitis, psoriatic arthritis and disease activity

## Abstract

Objective: Since obesity has been associated with a higher inflammatory burden and worse response to therapy in patients with chronic inflammatory rheumatic diseases (CIRD), we aimed to confirm the potential association between body mass index (BMI) and disease activity in a large series of patients with CIRDs included in the Spanish CARdiovascular in rheuMAtology (CARMA) registry. Methods: Baseline data analysis of patients included from the CARMA project, a 10-year prospective study of patients with rheumatoid arthritis (RA), ankylosing spondylitis (AS), and psoriatic arthritis (PsA) attending outpatient rheumatology clinics from 67 Spanish hospitals. Obesity was defined when BMI (kg/m^2^) was >30 according to the WHO criteria. Scores used to evaluate disease activity were Disease Activity Score of 28 joints (DAS28) in RA, Bath Ankylosing Spondylitis Disease Activity Index (BASDAI) in AS, and modified DAS for PsA. Results: Data from 2234 patients (775 RA, 738 AS, and 721 PsA) were assessed. The mean ± SD BMI at the baseline visit were: 26.9 ± 4.8 in RA, 27.4 ± 4.4 in AS, and 28.2 ± 4.7 in PsA. A positive association between BMI and disease activity in patients with RA (β = 0.029; 95%CI (0.01–0.05); *p* = 0.007) and PsA (β = 0.036; 95%CI (0.015–0.058); *p* = 0.001) but not in those with AS (β = 0.001; 95%CI (−0.03–0.03); *p* = 0.926) was found. Disease activity was associated with female sex and rheumatoid factor in RA and with Psoriasis Area Severity Index and enthesitis in PsA. Conclusions: BMI is associated with disease activity in RA and PsA, but not in AS. Given that obesity is a potentially modifiable factor, adequate control of body weight can improve the outcome of patients with CIRD and, therefore, weight control should be included in the management strategy of these patients.

## 1. Introduction

Obesity constitutes a major epidemic, in particular in developed countries. It affects around 35% of the general population according to the World Health Organization (WHO) [1]. In Spain, it reaches 21.6% of the global population, according to data from the Nutritional Study of the Spanish Population (ENPE-2015) [2]. Some studies suggest that there is a higher prevalence of obesity in individuals with rheumatoid arthritis (RA), psoriatic arthritis (PsA) [3,4,5], and ankylosing spondylitis (AS) [6].

A number of studies have confirmed that adipose tissue is a metabolically active organ, representing an important source of inflammatory mediators, known as adipokines or adipocytokines. They promote a pro-inflammatory state in obese subjects, establishing obesity as a low-grade inflammatory disease [7,8].

Higher body mass index (BMI) values are negatively associated with anti-TNF drug levels in plasma and, as a consequence, obesity has been associated with higher disease activity rates and worse response to therapies [9,10,11,12]. The relationship between obesity and disease activity has been reflected in some studies in patients with RA and PsA. Regarding AS, this relationship is less clear. However, some studies suggest a possible link between disease activity in patients with AS and excess fatty tissue [13,14]. In this regard, higher BMI values have been associated with lower response rates mainly to infliximab (IFX) [15] and other anti-TNF agents in patients with spondyloarthritis [16,17].

Taking all these considerations into account, the purpose of the present study was to confirm the potential association between BMI and disease activity in a large series of patients with chronic inflammatory rheumatic diseases (CIRD) included in the Spanish CARdiovascular in rheuMAtology (CARMA) registry.

## 2. Patients and Methods

### 2.1. Study Design

The CARdiovascular in rheuMAtology (CARMA) project is a prospective 10-year follow-up cohort study designed to determine the risk of cardiovascular mortality in patients with CIRDs compared to a cohort of patients without CIRD [18]. For the present study, a cross-sectional analysis of the initial visit has been performed to determine the relationship between disease activity and BMI at the baseline visit (time of recruitment). 

### 2.2. Patient Recruitment

All Spanish public hospitals (university and general hospitals) with Rheumatology Units included in the Spanish Rheumatology Society (Sociedad Española de Reumatología- SER) database, which includes more than 90% of the country’s hospitals, were invited to participate in the registry. Finally, 67 institutions (63.2% of all centers contacted) were included in the project. Overall, the patients recruited for the study were 2234. They attended rheumatology outpatient clinics at tertiary or secondary care centers between July 2010 and January 2012. Patients were included in the registry if they were older than 18 years and met at least 4 criteria of the American College of Rheumatology (ACR) 1987 for RA [19], the modified New York criteria for definite AS [20] or, in the case of PsA, the Moll and Wright criteria [21].

Information regarding the sample size and the baseline characteristics of the recruited participants, patients, and controls has previously been described by Castañeda et al. [18]. The study protocol was performed according to the principles of the Helsinki Declaration and it was approved by the Ethics Committee for Clinical Research of Lugo, Galicia (Spain), and subsequently also in each participant center (protocol number: 2009/077).

### 2.3. Variables and Operative Definitions

All included patients were evaluated on a continuous and systematic basis. To verify the quality of the information, an on-site evaluation of the follow-up data was performed randomly in 15% of the selected patients. The primary variable studied was the disease activity, which was measured through the Disease Activity Score of 28 joints (DAS28) [22] in the baseline visit for patients with RA. In the case of PsA, it was assessed through modified DAS28 [23], and by Bath Ankylosing Spondylitis Disease Activity Index (BASDAI) in AS patients [24]. The explanatory variable was the BMI measured in kg/m^2^.

Potential confounding factors included socio-demographic factors (such as age, sex, and education level), disease-specific variables (rheumatoid factor (RF), anti-citrullinated peptide antibodies (ACPA), positivity for HLA-B27, erythrocyte sedimentation rate (ESR, mm/h) by Westergren method, C-reactive protein (CRP, mg/L)), DAS28-ESR, modified DAS for PsA, Health Assessment Questionnaire (HAQ, from 0 to 3), Bath Ankylosing Spondylitis Disease Activity Index (BASDAI, from 0 to 10) and Bath AS Functional Index (BASFI, from 0 to 10), duration of disease, and administered therapies. The variables of physical activity and smoking are collected through an interview with the patient. Physical activity was classified into three categories: mild, moderate, and high physical activity depending on the hours of exercise per week. Height was measured in the patients at the beginning of the study (visit 0). Data on medication are collected by reviewing the medical history (see supplementary material of [18]).

### 2.4. Statistical Analysis

A baseline descriptive analysis was performed. Numerical variables with a normal distribution were expressed as mean and standard deviation (SD). Non-normally distributed variables were shown as the median and interquartile range (IQR, P_25_–P_75_). The absolute and relative frequencies of the qualitative variables were also calculated.

Analyses of the main demographic and clinical variables stratified by the type of disease were done. Furthermore, a stratified analysis was performed through linear regression for each group according to activity indices and BMI (kg/m^2^). Moreover, socio-demographic characteristics and clinical features were analyzed.

To study the relationship between disease activity and BMI (kg/m^2^), three explanatory models of linear regression were constructed, one for each disease, calculating the beta (β) coefficient, with 95% confident interval (CI) and adjusting for potential confounding factors.

Data management and statistical analysis were centralized at the Research Unit of the Spanish Rheumatology Society. All analyses were performed using Stata 13.1 copyright 1985–2013 StataCorp LP (Lakeway Drive College Station, Texas, USA).

## 3. Results

### 3.1. General Characteristics of the Patients

The total number of patients included in the study was 2234 (775 RA, 738 AS, 721 PsA). Socio-demographic and clinical characteristics of the patients included have been described in previous studies [18] (Table 1), with an increased prevalence of women in the RA group (74.9%) and a greater percentage of men in AS group (72.9%), while sex distribution was almost similar in PsA patients. The average age in patients with RA was higher (57.1 years) than in the other two groups. By contrast, the average BMI and the frequency of obesity were higher in patients with PsA. Furthermore, diabetes was more frequent in PsA patients. The percentage of active smokers was higher in the AS group (34.4%). Remarkably, the majority of CIRD patients included in the study had low disease activity at the time of inclusion in the registry because patients were followed up in outpatient clinics of Rheumatology, mostly under tight control conditions of management of the disease. In keeping with that, the percentage of patients receiving biologic therapy ranged between 41.4% in RA up to 47.2% in AS (Table 1).

### 3.2. Association between Body Mass Index and Disease Activity

The effect of BMI on disease activity, estimated as β-coefficient by multivariate linear regression, is shown in Table 2. We found a positive association between BMI and disease activity in patients with RA (β-coefficient: 0.029; 95% CI: 0.01–0.05; *p* = 0.007) and in those with PsA (β-coefficient: 0.036; 95% CI: 0.015–0.058; *p* = 0.001). By contrast, there was no significant association between BMI and disease activity in patients with AS (β-coefficient: 0.001; 95% CI: −0.026–0.03; *p* = 0.926). When categorizing BMI variable as normal weight (BMI < 25), overweight (BMI: 25–30), and obesity (BMI > 30), similar significance was maintained in patients with RA (B-coeff: 0.32; 95% CI:0.067–0.571; *p* = 0.013), while in PsA, the significance remained only at limited values (B-coeff: 0.27; 95% CI:−0.0026–0.537; *p* = 0.05), probably due to the reduction in the number of patients in each group, maintaining non-significant values in patients with AS (B-coeff: 0.073; *p* = 0.662).

In patients with RA, female sex (β-coefficient: 0.546; 95% CI: 0.316–0.775; *p* < 0.001) and rheumatoid factor status (seropositivity for RF) (β-coefficient: 0.328; 95% CI: 0.106–0.549; *p* = 0.004) also showed a positive association with disease activity, while physical activity revealed a negative association with disease activity (β-coefficient: −0.280; 95% CI: −0.479–(−0.081); *p* = 0.006) (Table 2).

Besides BMI, female sex (β-coefficient: 0.720; 95% CI: 0.524–0.916; *p* < 0.001), Psoriasis Area Severity Index (PASI) (β-coefficient: 0.038; 95% CI: 0.012–0.066; *p* = 0.005), and enthesitis (β-coefficient: 0.256; 95% CI: 0.199–0.313; *p* < 0.001) were also positively associated with disease activity in PsA (Table 2).

As observed in RA and PsA, female sex was also associated with disease activity in patients with AS (β-coefficient: 0.565; 95% CI: 0.299–0.832; *p* < 0.001) (Table 2).

## 4. Discussion

The main findings of this study support an association of BMI with disease activity in RA and PsA but not with AS. In addition, specific features of each CIRD were associated with disease activity. It was the case for RF status in patients with RA and PASI and the presence of enthesitis in those with PsA. Central obesity is one of the components of the metabolic syndrome that is frequently observed in patients with CIRD, in particular in those with RA and PsA [25,26,27].

The reason for the absence of the association of BMI with disease activity in patients with AS from the CARMA project is unknown. With respect to this, AS patients included in the CARMA cohort had low disease activity and almost half of them were undergoing biologic therapy. In this regard, it may be of potential interest to highlight that the anti-TNF monoclonal antibody-IFX was more frequently used in AS (18.3%) than in RA (6.2%) and PsA (10.1%). Given that IFX is a biological agent that is administered adjusted to the weight of the subject, we wondered if this could lead to an optimal response and less disease activity in this group of patients. According to this possibility, Micheroli et al. reported that axial spondyloarthritis obese patients treated with IFX had a better response compared to those treated with other anti-TNF agents not adjusted to corporal weight [16]. Another possible explanation may be that the implication of the metabolic syndrome and, consequently, of the proinflammatory adipokines in AS appears to be less important than that played in the other two CIRDs. On the other hand, our data also supported the potential beneficial effect of regular physical activity and weight loss on the control of the underlying disease in individuals with RA.

Obesity and overweight have an important role in the development of psoriasis and PsA. In a cohort study among 75,395 individuals with psoriasis (43% male, mean age of 52 years, and mean follow-up of 5 years) conducted in the UK, 976 developed PsA (incidence rate of 26.5 per 10,000 person-years) [28]. The PsA incidence rates augmented with increasing BMI. Compared to psoriasis patients with BMI < 25 kg/m^2^, the relative risk for developing PsA was 1.09 (0.93–1.28) for BMI from 25.0 to 29.9, 1.22 (1.02–1.47) for BMI from 30.0 to 34.9, and 1.48 (1.20–1.81) for BMI ≥ 35 [28]. In another study including 89,049 participants from the Nurses’ Health Study II followed over a 14-year period, 146 incident PsA cases were identified during 1,231,693 person-years of follow-up. Among all participants, BMI was associated with an increased risk of incident PsA [29]. There was a graded positive association between weight change from age 18 years, measures of central obesity, and risk of PsA. The analysis among participants developing psoriasis during the follow-up revealed a similar association, indicating an increased risk of PsA associated with obesity among patients with psoriasis [29]. These two studies suggest that obesity is associated with an increased risk of incident PsA and supports the importance of weight control among psoriatic patients who often suffer from metabolic syndrome and obesity. As previously reported by other authors [17,30,31,32,33], obese patients from the CARMA cohort were treated more commonly with biologic therapies than normal-weight individuals.

Finally, besides its direct effect on inflammation through increased proinflammatory cytokines secretion, obesity can also influence treatment response by modifying pharmacokinetics of biologic antirheumatic drugs. In fact, population studies have identified high body weight as a risk factor for increased clearance of anti-TNF agents, resulting in shorter half-life and lower serum drug concentrations [34]. By contrast, there is strong clinical evidence that while TNF blockers may be less effective in obese patients, the efficacy of T cell co-stimulation inhibition, B-cells depletion, and IL-6 blocking seems not to be negatively influenced by the amount of body fat [35].

Our study has some strengths. It includes an important national multicenter cohort of patients with RA, AS, and PsA prospectively followed up at the outpatient clinics to detect the incidence of cardiovascular events over 10 years of follow-up, being the first study in our country that tries to establish an association between BMI and inflammatory activity in patients with these three diseases. However, it has also some limitations. Many of the patients included in this study had low disease activity at the time of recruitment. In fact, our patients were under a tight control strategy and a high number of them were undergoing biologic therapy at recruitment. This management may have had decreased some of the disease activity parameters used, thus interfering in the results. In this regard, obesity is a modifiable factor in our clinical practice that not only plays an important role in cardiovascular risk and morbidity but its control would also imply better management of the inflammatory disease and better efficiency of the treatments, especially biological agents, used in these patients.

In summary, a relationship between BMI and disease activity was observed in patients with CIRD from the CARMA cohort, especially in patients with RA and PsA. This finding could not be demonstrated in patients with AS, where the proportion of patients treated with biologic therapy was higher, which could have modified the results obtained. 

Adequate control over body weight may improve the outcome of patients with inflammatory joint diseases and, therefore, weight control should be included in the strategy of management of these patients.

## Figures and Tables

**Table 1:** Main demographic, lifestyle, and clinical characteristics of the patients included in the study.

Variables	Rheumatoid Arthritis (*n* = 775)	Ankylosing Spondylitis (*n* = 738)	Psoriatic Arthritis (*n* = 721)	*p*
Age at inclusion, years, mean (SD)	57.1 (12.3)	48.1 (11.7)	51.8 (12.0)	<0.001
Age at the beginning of disease, years, mean (SD)	45.8 (13.4)	29.7 (11.8)	39.5 (13.3)	<0.001
Female sex, *n* (%)	581 (75.0)	200 (27.1)	327 (45.4)	<0.001
BMI, kg/m^2^, mean (SD)	26.9 (4.8)	27.4 (4.4)	28.2 (4.7)	0.005
Physical activity	462 (66.1)	453 (69.7)	409 (62.9)	0.035
Smoking				
Current	172 (24.4)	224 (34.1)	136 (20.6)	<0.001
Past	188 (26.7)	212 (32.3)	216 (32.7)	
Never	345 (48.9)	221 (33.6)	308 (46.7)	
Ethnicity				
Caucasic	679 (96.6)	643 (97.9)	654 (99.2)	<0.001
Hispanic	20 (2.8)	6 (0.9)	2 (0.3)	
Other	4 (0.1)	8 (1.2)	3 (0.5)	
Educational level				
Basic	59 (8.5)	25 (3.8)	32 (4.9)	<0.001
Primary	367 (52.6)	257 (39.4)	269 (41.2)	
Secondary	171 (24.5)	199 (30.5)	189 (28.9)	
University	101 (14.5)	172 (26.3)	163 (25.0)	
Obesity (BMI ≥ 30), *n* (%)	180 (23.2)	186 (25.2)	209 (29.1)	<0.001
Disease duration, years	8.0 (3.0–14.0)	15.0 (8.0–26.0)	9.0 (4.0–16.0)	<0.001
DAS28-ESR, mean (SD)	3.2 (1.2)	-	3.0 (1.3)	0.005
BASDAI (0–10), median (P_25_–P_75_)	-	3.5 (1.7–5.3)	-	-
HAQ (1–3), median (P_25_–P_75_)	0.5 (0.1–1.1)	-	0.4 (0.0–0.9)	<0.001
BASFI (0–10), median (P_25_–P_75_)	-	3.1 (1.3–5.2)	-	-
ESR, mm/1st h, median (P_25_–P_75_)	17.0 (9.0–29.0)	10.0 (6.0–21.0)	12.0 (6.0–21.0)	<0.001
CRP, mg/L, median (P_25_–P_75_)	3.1 (1.2–8.0)	3.6 (1.6–8.9)	2.9 (1.4–6.1)	0.001
ACPA positive (%)	420 (59.6)			-
RF positive (%)	541 (76.7)			-
HLA-B27 positive (%)		498 (75.8)		
Onicopathy, *n* (%)			254 (38.5)	-
PASI, median (P_25_–P_75_)			0.6 (0.0–2.1)	-
Enthesitis, median (P_25_–P_75_)			0.0 (0.0–1.0)	-
Conventional DMARDs, *n* (%)	619 (87.8)	209 (31.8)	491 (74.4)	<0.001
Biologic DMARDs, *n* (%)	292 (41.4)	310 (47.2)	278 (42.1)	0.068
GC (ever treated), *n* (%)	320 (45.4)	54 (8.2)	117 (17.7)	<0.001

BMI: body mass index; DAS28-ESR: Disease Activity Score using 28 joints-erythrocyte sedimentation rate; BASDAI: Bath Ankylosing Spondylitis (AS) Disease Activity Score; HAQ: Health Assessment Questionnaire; BASFI: Bath AS Functional Index; CRP: C-reactive protein; ACPA: anti-cyclic citrullinated peptide antibodies; RF: rheumatoid factor; HLA-B27: histocompatibility antigen; PASI: Psoriasis Area Severity Index; DMARD(s): Disease-modifying antirheumatic drugs; GC: glucocorticoids. Data expressed as median (P_25_–P_75_) unless specified. Dichotomous variables are expressed as n and percentages (%); SD: standard deviation.

**Table 2:** Association between disease activity and epidemiologic and clinical features in patients with CIRD from the CARMA cohort. Multivariate analysis.

Variables	Rheumatoid Arthritis (*n* = 775)			Ankylosing Spondylitis (*n* = 738)			Psoriatic Arthritis (*n* = 721)		
	β	95% CI	*p*-Value	β	95% CI	*p*-Value	β	95% CI	*p*-Value
BMI (kg/m^2^)	0.029	(0.01, 0.05)	0.007	0.001	(−0.03, 0.03)	0.926	0.036	(0.015, 0.058)	0.001
Age at inclusion	−0.003	(−0.01, 0.004)	0.312	−0.005	(−0.02, 0.005)	0.348	−0.003	(−0.011, 0.004)	0.325
Sex (reference, male)	0.545	(0.316, 0.775)	<0.001	0.565	(0.299, 0.832)	<0.001	0.720	(0.524, 0.916)	<0.001
Ethnicity (reference, Caucasic)									
Hispanic	−0.234	(−0.810, 0.343)	0.426	0.422	(−0.788, 1.633)	0.493	1.077	(−0.529, 2.684)	0.188
Other	−0.789	(−1.986, 0.406)	0.195	0.119	(−1.095, 1.334)	0.847	−1.370	(−2.977, 0.237)	0.095
Physical activity (reference, no)	−0.280	(−0.479, −0.081)	0.006	0.0009	(−0.260, 0.262)	0.994	−0.100	(−0.296, 0.095)	0.313
Educational level (ref., Primary)									
Primary	−0.136	(−0.481, 0.201)	0.437	0.374	(−0.254, 1.004)	0.242	−0.358	(−0.80, 0.083)	0.112
Secondary	−0.350	(−0.732, 0.035)	0.075	0.332	(−0.311, 0.975)	0.311	−0.538	(−0.999, −0.767)	0.022
University	−0.137	(−0.551, 0.275)	0.513	0.321	(−0.341, 0.984)	0.341	−0.540	(−1.009, −0.075)	0.024
Smoking (ref., current smokers)									
Past smokers (>1 year)	0.102	(−0.166, 0.370)	0.455	−0.043	(−0.330, 0.242)	0.765	0.081	(−0.188, 0.352)	0.552
Never smokers	0.081	(−0.157, 0.319)	0.504	0.010	(−0.279, 0.300)	0.942	−0.049	(−0.299, 0.200)	0.696
Rheumatoid factor + (reference, no)	0.328	(0.106, 0.549)	0.004	NA			NA		
HLA-B27 + (reference, no)	NA			−0.099	(−0.376, 0.178)	0.484	NA		
BASFI	NA			0.656	(0.608, 0.706)	<0.001	NA		
PASI	NA			NA			0.038	(0.012, 0.066)	0.005
Enthesitis	NA			NA			0.256	(0.199, 0.313)	<0.001
Onicopathy (reference, no)	NA			NA			0.155	(−0.034, 0.346)	0.109
Conventional DMARDs (ref., no)	−0.059	(−0.341, 0.223)	0.680	0.028	(−0.023, 0.286)	0.829	0.193	(−0.029, 0.415)	0.089
Biologic DMARDs (reference, no)	0.095	(−0.10, 0.29)	0.338	−0.431	(−0.665, −0.198)	<0.001	−0.353	(−0.553, −0.154)	0.001
Glucocorticoids (reference, no)	0.187	(0.001, 0.373)	0.049	0.373	(−0.054, 0.802)	0.087	0.326	(0.083, 0.570)	0.008

BASFI: Bath Ankylosing Spondylitis (AS) Functional Index; BMI: body mass index; CARMA: CARdiovascular in RheuMAtology; CIRD: chronic inflammatory rheumatic diseases; DMARD(s): disease-modifying anti-rheumatic drugs; HLA-B27: Human leucocyte antigen B27; PASI: Psoriasis Area Severity Index. Data are expressed as β-coefficients (95% CI) and *p*-values. NA: not applicable.

## CARMA Project Collaborative Group

Members of the CARdiovascular in rheuMAtology Project Collaborative Group include: José Antonio Pinto-Tasende, Elena Alonso Blanco Morales, J. Carlos Fernández López, Natividad Oreiro Villar, Antonio Atanes Sandoval, Francisco J. Blanco García (Complejo Hospitalario A Coruña, Xubias de Arriba, A Coruña); Cayetano Alegre De Miquel, María J. González Fernández, Ramón Huguet Codina, Beatriz Yoldi, Mercedes Ramentol (Instituto Dexeus, Barcelona); Sara Marsal Barril, María Lopez Lasanta, Susana Anton, Estefanía Quesada (Hospital Universitari Vall d’Hebron, Barcelona); Martina Steiner, Santiago Muñoz, Tatiana Cobo (Hospital Infanta Sofía, Madrid); Maria del Puerto Moreno Gil, Fernando Gamero, José García Torón, (Hospital S. Pedro de Alcántara, Cáceres); Antonio Juan Mas, Pilar Espino, Inmaculada Ros, Mónica Ibáñez, Claudia Murillo, Catalina Melia Mesquida, Ana Paula Cacheda (Hospital Son Llatzer, Palma de Mallorca); Jesús Tornero Molina (Hospital Univ. de Guadalajara); Raimon Sanmartí, Horacio Berman, Sonia Cabrera, Virginia Ruiz, Raúl A. Castellanos Moreira, Sebastián C. Rodríguez García (Hospital Clinic i Provincial, Barcelona); Benjamín Fernández Gutiérrez, Luis Rodríguez Rodríguez, Lydia Abasolo, Oscar Fontseré Patón (Hospital Clínico Univ. San Carlos, Madrid); José M. Pina, Dolores Fábregas Canals (Hospital de Barbastro, Huesca); Montserrat Romera, Joan M. Nolla, (Hospital Univ. de Bellvitge, Barcelona); Miriam García Arias, Mirem Uriarte Ecenarro, Jesús A. García Vadillo, Rosario García de Vicuña (Hospital Univ. de La Princesa, Madrid); Antonio Fernández Nebro, María Ángeles Belmonte López, Sara Manrique Arija, Inmaculada Ureña, María V. Irigoyen, Virginia Coret Cagigal (Hospital Regional Univ. Carlos Haya, Málaga); José Santos Rey, Daniel Pielfort Garrido, Ángel M. García Aparicio, Rebeca Belmonte Gómez, Pastora Granados Bautista, Azucena Hernández Sanz (Hospital Virgen de la Salud, Toledo); Javier Bachiller (Hospital Ramón y Cajal, Madrid); Francisco J. Manero, Fernando Jimenez Zorzo, Eugenio Giménez Úbeda, Jesús Marzo Gracía, Chesús Beltrán Audera, Marta Medrano, Ángela Pecondón (Hospital Univ. Miguel Servet, Zaragoza); Celia Erausquin, Soledad Ojeda, Juan C. Quevedo, Félix Francisco, Carlos Rodríguez Lozano (Hospital Dr. Negrín, Las Palmas de Gran Canaria); Indalecio Monteagudo, Francisco J. López Longo, Delia Gerona, Carlos González Fernández (Hospital Gregorio Marañón, Madrid); Javier del Pino, Ana Turrión (Hospital Univ. de Salamanca); Alfonso Corrales, Diana Prieto-Peña (Hospital Univ. Marqués de Valdecilla, Santander); José M. Senabre, José C. Rosas (Hospital Marina Baixa, Alicante); Isabel Rotés, Estefanía Moreno, Laura López Vives, Alba Erra, Dolors Grado (Hospital San Rafael, Barcelona); Javier Calvo, Amalia Rueda (Hospital General Universitario, Valencia); Ingrid Möller, Isabel Rodríguez (Instituto Poal, Barcelona); Carmen Barbadillo (Hospital Univ. Puerta de Hierro, Madrid); Enrique Raya, Pilar Morales, Ana Nieto, Inmaculada Jiménez, Cesar Magro, José Luis Rosales Alexander (Hospital Clínico Univ. San Cecilio, Granada); Ana Ruibal Escribano (Hospital Santiago Apóstol, Vitoria-Gasteiz); Sergio Ros Expósito (Hospital de Viladecans, Barcelona); Enrique Júdez Navarro, Manuela Sianes Fernández, María A. García Morales, Isabel Labiano Bastero, Gloria García Consuegra, Natalia Palmou (Hospital General de Albacete); Silvia Martínez Pardo, Manel Pujol, Elena Riera Alonso, Georgina Salvador (Hospital Mutua Terrassa, Terrassa); Beatriz González Alvarez, Alberto Cantabrana (Hospital Ntra. Sra. de Candelaria, Santa Cruz de Tenerife); Sagrario Bustabad, Esmeralda Delgado (Hospital Univ. de Canarias, La Laguna, Tenerife); Alejandro Muñoz, Sergio Rodriguez Montero, Luis María Jimenez (Hospital Univ. de Valme, Sevilla); Teresa Gonzalez Hernandez (Instituto Provincial de Rehabilitación, Madrid); Emilio Giner Serret, Carla Lannuzzelli Barroso (Hospital Obispo Polanco, Teruel); Laura Cebrián Méndez, María Teresa Navío (Hospital Infanta Leonor, Madrid); Cristina Fernández Carballido, Teresa Pedraz Penalva, Elisabet Berzosa Sola (Hospital General de Elda, Alicante); Encarnación Pagán, Pablo Mesa del Castillo (Hospital Los Arcos, Murcia); Cristina Clara Macía Villa, Ana Cruz Valenciano (Hospital Severo Ochoa, Madrid); Julio Sánchez, María Galindo, Javier García González (Hospital Univ. 12 de Octubre, Madrid); Eduardo Collantes, Desireé Ruiz, Jerusalem Calvo, Laura Bautista (Hospital Univ. Reina Sofía, Córdoba); Gema Bonilla (Hospital Univ. La Paz, Madrid); Antonio López Meseguer (Hospital Gutiérrez Ortega, Valdepeñas, Ciudad Real); María J. Moreno Martínez; María D. Beteta Fernández, Luis F. Linares (Hospital Virgen de la Arrixaca, Murcia); María L. González Gómez (Hospital del Escorial, Madrid); Natalia A. Rivera, Olaia Fernández Berrizbeitiar (Hospital de Basurto, Bilbao); Manel Riera, Yolanda María León (Hospital Dos de Maig, Barcelona); Jordi Fiter, Julia Fernández Melón, Luis Espadaler (Hospital Univ. Son Espases, Palma de Mallorca); Joaquín Belzunegui; Inmaculada Bañe Gil (Hospital de Donostia, Donostia); María Bonet, Estefania Moreno Ruzafa (Hospital Comarcal de L’Alt Penedés, Vilafranca del Penedés, Barcelona); Francisco J. Navarro Blasco, J. Antonio González (Hospital General Univ. de Elche, Alicante).
